# Editorial: Technological advances in psychiatric treatments: a focused exploration of human-computer interaction and human factors in digital therapeutics

**DOI:** 10.3389/fpsyt.2026.1814653

**Published:** 2026-03-20

**Authors:** Samuel Ridout, Alaa Abd-alrazaq, Khaldoon Dhou

**Affiliations:** 1 Department of Psychiatry, Kaiser Permanente, San Jose, CA, United States; 2 Weill Cornell Medicine – Qatar, Doha, Qatar; 3 Texas A&M University Central Texas, Killeen, TX, United States

**Keywords:** digital, engagement, implementation, mental health, psychiatry, technology

## Introduction

The landscape of mental health research and treatment is changing quickly with the advent of digital tools designed to augment or, in some cases, replace standard models of patient care. As social acceptance of mental health treatment increases, so does the demand for services. In the future, the landscape of population management will necessarily include digital therapeutics (DTx) as core components of patient evaluation and management. This is not a far-fetched idea borne of science fiction; it is the reality of the next phase of mental health research and treatment, and the central premise of this Research Topic, *“Technological Advances in Psychiatric Treatments: A Focused Exploration of Human-Computer Interaction (HCI) and Human Factors in Digital Therapeutics.”* The Research Topic begins with an urgent observation: digital tools in psychiatry are proliferating rapidly; however, the evidence base for how, when, and for whom they work in real-world care remains uneven. To close this gap, we must examine not only clinical outcomes, but also the **interaction**—the usability, workflow fit, safety, trustworthiness, and engagement dynamics that determine whether a tool can become a therapy at all (1).

Why HCI is not an accessory, but rather, the mechanism

DTx is “evidence-based, clinically evaluated software to treat, manage, or prevent diseases” (2). In practice, that means every therapeutic claim is mediated by human behavior at an interface: the decision to open an app, the ease of navigating a module, the comfort of a headset, the perceived credibility of an AI explanation, and the way a feature aligns (or clashes) with clinical workflows. The Research Topic that anchors this special edition explicitly argues that focusing on HCI and human factors can deepen our understanding of DTx use and optimize design for better outcomes, particularly as telehealth and AI accelerate adoption pressures across psychiatric care (1).

The seven articles in this Research Topic take this idea seriously. They span modalities—virtual reality, serious games, mobile applications, and explainable AI—and they span contexts—self-guided skill building, psychosocial care integrated with oncology, neurorehabilitation after stroke, schizophrenia cognition, and opioid treatment programs. However, a shared storyline runs through all of them: effective digital therapeutics are rarely “set-and-forget.” They are co-produced by design choices and human use in context (1, 2).

Training the micro-skills that make therapy possible

In psychotherapy, progress often hinges on micro-skills: noticing a thought, labeling an emotion, tolerating discomfort, and practicing a new response. Yang and colleagues target one of the most foundational of these skills—identifying negative automatic thoughts—using a virtual reality, self-guided training sequence. Their mixed-methods feasibility study emphasizes acceptability and user experience alongside performance on skill-linked measures, directly acknowledging a core HCI truth: if the experience is disorienting, fatiguing, or unclear, the “therapy” cannot be delivered as intended. In their account, VR is not merely a novel wrapper; it is a design space for scaffolding cognitive restructuring in ways that standard digital formats may struggle to achieve.

A different route to the same destination—strengthening psychological functioning through digitally mediated experience—appears in Liu and colleagues’ trial of group computer magnanimous therapy (GCMT) in advanced lung cancer. Their randomized study pairs psychological outcomes with magnetic resonance spectroscopy markers, linking measured change to both lived experience and potential mechanistic correlates. The clinical population and compressed intervention window underscore a human factors challenge that is often underestimated: when users are fatigued, distressed, or medically burdened, design constraints tighten. A “good” intervention becomes one that fits the realities of attention, stamina, and meaning-making in illness—not simply one that is theoretically sound.

When “engagement” is engineered, not wished for

Rehabilitation science has long grappled with adherence. Oliveri and colleagues extend this conversation to digital design by evaluating **prism adaptation combined with serious games** to improve visual-constructive abilities in stroke patients. Notably, their study asks which serious games best predict improvements and whether gains generalize to daily living skills—questions that treat “game choice” and interaction patterns as candidate active ingredients rather than decorative features. This is HCI as therapeutic theory: interface components are not neutral; they can shape learning, transfer, and functional impact.

In opioid treatment programs, the engagement problem is even more immediate: missed visits and disengagement can carry lethal risk. Palacios and colleagues analyze the implementation of Recovery Connect, a mobile app integrated into medication treatment for opioid use disorder across dozens of clinics. Their focus on acceptability, clinician-patient communication, early engagement behaviors, and associations with retention highlights a pragmatic reality: in large systems, the most meaningful outcomes may depend less on content ideals and more on the *interaction patterns* a tool enables—messaging workflows, self-monitoring routines, and timely feedback loops that keep people connected to care.

## Do we always need a human in the loop?

Digital mental health has often relied on human support to drive adherence—coaching, check-ins, and accountability. However, support adds cost and complexity, limiting scale. Saar, Brandes, and Baumel confront this tradeoff head-on by asking whether human support adds value when an intervention is already built with “therapeutic persuasiveness” design features. Using propensity score matching across comparable cohorts in a digital parenting program, they compared engagement patterns and clinical outcomes between human-supported and self-directed formats. This study contributes to the field by reframing a central implementation question as an empirical design problem: Which supportive functions can be reliably built into the interface, and which require human judgment?

## Cognitive impairment in schizophrenia: a clinical north star for DTx design

Sun and colleagues’ opinion piece on digital therapeutics for cognitive impairments associated with schizophrenia provides a crucial clinical anchor. Cognitive deficits remain a core driver of functional disability, and current treatment options have notable limitations. The authors argue that digitally enabled approaches may offer paths forward while implicitly raising the HCI bar for this population: tools must be usable under motivational and cognitive constraints, feasible outside of specialized settings, and engaging enough to sustain practice. In other words, schizophrenia cognition is not only a treatment target; it is a stress test for human-centered DTx design.

## Trustworthy AI: when explanation is part of the interface

Finally, the MATRIX study by Ramnani, Roy, and Sheth introduces a real-time diagnostic support system that uses natural language interaction with PHQ-9 content and produces explainable, attributable reasoning (including clinically legible mappings such as standardized concept identifiers). Here, human factors are not secondary—they are the point. Diagnostic support that cannot be inspected, understood, and appropriately calibrated by clinicians risks either disuse or overreliance. MATRIX positions interpretability as a design requirement for safe collaboration between clinician and algorithm.

## What this special edition contributes: a clearer map of the field’s missing middle

Taken together, these seven articles highlight a “missing middle” in digital psychiatry research: the space between efficacy and implementation (real-world delivery). In this space, HCI variables such as usability, clinician trust, and workflow fit determine whether interventions are usable, trusted, and durable enough to matter. The Research Topic’s framing emphasizes this gap explicitly—pointing to proliferation without sufficient evidence and calling for interdisciplinary, HCI-informed work to refine designs and functions for therapeutic outcomes (1).

## Looking forward: the next questions

If digital therapeutics are to mature into a reliable part of psychiatric care, the field must move beyond asking whether tools “work” in general and toward asking the following: *Which interaction ingredients drive change, for whom, and under what constraints?* This includes standardizing **meaningful engagement metrics** (beyond logins), designing for diverse cognitive and clinical needs, evaluating safety as a first-class outcome, and building transparent AI systems that clinicians can validate rather than merely consult. Specifically, these studies identify several critical interaction ingredients:

- Therapeutic Persuasiveness: Integrating adherence support directly into the software design.
- Cognitive Scaffolding: Using immersive environments, such as VR, to guide complex psychological skills.
- Explainable Reasoning: Ensuring that AI diagnostics provide “clinically relevant” evidence to earn provider trust.
- Contextual Adaptability: Designing specifically for the attention and stamina constraints of distressed patients.

Ultimately, the promise of DTx will not be realized by technology alone. It will be realized when interfaces are crafted as carefully as interventions and when human factors science is treated as core clinical science. While HCI focuses on the immediate exchange between the user and the interface, human factors science addresses the broader systemic fit and workflow integration required for clinical viability. This special edition is an invitation to build that future deliberately.
